# Effect of pyrogallol on the physiology and biochemistry of litchi fruit during storage

**DOI:** 10.1186/1752-153X-7-19

**Published:** 2013-01-30

**Authors:** Guoxing Jing, Hua Huang, Bao Yang, Jianrong Li, Xiaolin Zheng, Yueming Jiang

**Affiliations:** 1 College of Food Science and Biotechnology, Zhejiang Gongshang University, Food Safety Key Lab of Zhejiang Province, Hangzhou, 310035, People’s Republic of China; 2 Key Laboratory of Plant Resources Conservation and Sustainable Utilization, South China Botanical Garden, Chinese Academy of Sciences, Guangzhou, 510650, People’s Republic of China; 3 School of Life Science and Technology, Zhanjiang Normal University, Zhanjiang, 524048, People’s Republic of China

**Keywords:** Litchi fruit, Pyrogallol, Physiology, Postharvest, Quality, Storage

## Abstract

**Background:**

Litchi (*Litchi chinensis* Sonn.) fruit are highly perishable and have a very short shelf life, easily turning brown and decaying. This study investigated the efficiency of pyrogallol, a catechin on the physiology and biochemistry in relation to storage life of litchi fruit.

**Results:**

Fruit were treated with pyrogallol at 1 mM and then stored at ambient temperature (25°C) or low temperature (4°C). Compared with control, pyrogallol significantly reduced pericarp browning and delayed the rotting of fruit day 4 at 25°C, and on day 30 at 4°C. The chemical treatment reduced respiration rate and the activities of peroxidase (POD) and polyphenol oxidase (PPO), and delayed the loss of membrane permeability. Pyrogallol increased the activity of phenylalanine ammonia-lyase (PAL), delayed the loss of anthocyanin and phenolics, and maintained high 2,2-diphenyl-1-picrlhydrazyl (DPPH) radical scavenging activity and reducing power. High performance liquid chromatograph (HPLC) analysis clearly indicated that treated fruit contained higher concentration of the four phenolic compounds procyanidin B1, (+)-catechin, (−)-epicatechin and (−)-epicatechin-3-gallate.

**Conclusions:**

The application of pyrogallol partially reducing pericarp browning and changed quality-related physiological activities and, thus, pyrogallol could have beneficial effects on pericarp browning and fruit decay control, and could be helpful for litchi fruit postharvest storage.

## Background

Litchi (*Litchi chinensis* Sonn.) is a subtropical fruit with attractive red pericarp and translucent white aril. However, the fruit rapidly lose their red color during storage and become less attractive in the market. Previous studies demonstrated that the loss of membrane integrity was involved in litchi pericarp browning [1]. The membrane deterioration results in cellular de-compartmentalization [2]. Accordingly, lipid peroxidation reduces membrane fluidity and increases membrane permeability [3], which leads to a mixing of enzymes and their substrates. Therefore, maintenance of membrane compartmentalization could be used to extend the storage life of litchi the fruit.

Polyphenol oxidase (PPO) and peroxidase (POD) are involved in the degradation of anthocyanins by catalyzing the oxidization of phenolic compounds [4,5]. The loss of cellular compartmentation during senescence caused a rapid degradation of red pigment promoted by PPO and/or POD, producing brown by-products [4]. It is proposed that litchi anthocyanins may first be hydorlysed to anthocyanidin, while some phenolic compounds such as 4-methylcatechol may accelerate anthocyanidin degradation in the presence of POD and PPO [4]. Thus, inhibition of PPO and POD activities might help to delay browning.

Reactive oxygen species (ROS) including superoxide, hydrogen peroxide, hydroxyl, peroxyl and alkoxyl, produced *in vivo* in living organisms, are associated with lipid peroxidation, leading to fruit browning [3]. Although almost all living organisms possess antioxidant defence and repair systems to protect against this injury, these are insufficient sometimes [6]. Fruit and vegetables contain different antioxidant compounds, such as ascorbic acid, tocopherol, glutathione and carotenoids, which may protect against ROS damage [7]. Thus, the application of antioxidant compounds could offer protection against damage and delay fruit browning. Pyrogallol is a catechin and is often used to investigate the role of O_2_^·-^ in biological systems. For example, it has been demonstrated that pyrogallol moiety is an active component of flavonoid for displaying high O_2_^·-^ scavenging activity because pyrogallol is much more efficient in scavenging O_2_^·-^ than catechol [8]. Unfortunately, little information is available on the role of pyrogallol in the storage life of fruit.

In the current study, an attempt has been made to investigate the effect of pyrogallol on litchi fruit during storage at ambient temperature (25°C) or low temperature (4°C). The experiment was planned to restrict the postharvest pericarp browning and microbial growth during storage and, thus, to maintain its sensory appeal the quality parameters such as physical, physiological, biochemical and antioxidant properties were also periodically evaluated during storage, in parallel.

## Results and discussion

### Disease incidence and pericarp browning

**Figure 1:**
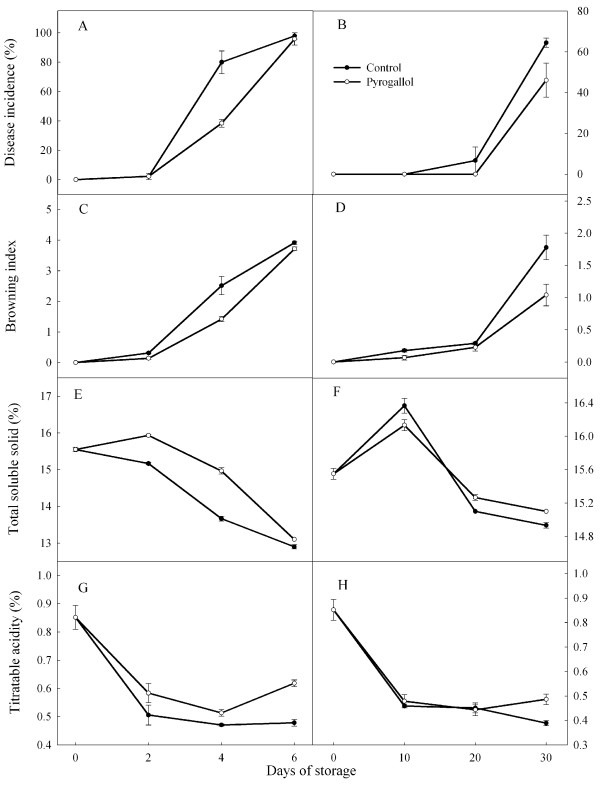
**Effect of pyrogallol on disease incidence and browning index, total soluble solids (TSS) and titratable acidity (TA) in ‘Huaizhi’ litchi fruit during storage at 25 (A, C, E and G) or 4°C (B, D, F and H).** Data are the means ± standard errors of 30 fruit for the disease incidence and browning index or 3 replicates for total soluble solids and titratable acidity. ● = control, and ○ = pyrogallol.

The incidences of disease and browning increased after 2 days at 25°C and after 20 days at 4°C (Figure 1). Overall fruit appearance (especially the red color) was better at 4°C than at 25°C. Pyrogallol reduced disease and browning on day 4 at ambient temperature and on day 30 at low temperature.

Litchis easily lose their red pigment after harvest, and become susceptible to fungi [9]. Jiang et al. [10] reported that NaHSO_3_ combined with HCl preserved the red colour. Zheng and Tian [11] found that oxalic acid delayed browning and suggested it could be used after harvest. Duan et al. [12] and Yi et al. [13] reported that pure oxygen and adenosine triphosphate (ATP) inhibited browning and prevented infection, but these treatments are not used commercially. Our study exhibited that pyrogallol improved the red colour of the fruit when stored for 4 days at ambient temperature or for 30 days at low temperature. Overall, the fruit were best with or without treatment for up to 2 days at 25°C, and up to 20 days at 4°C.

### Total soluble solids and titratable acidity

Average values of total soluble solids (TSS) increased slightly during storage at 4×°C and 25°C, and then decreased (Figure 1). In contrast, average values of titratable acidity (TA) tended to decrease during storage and were similar at the two temperatures. Average values of TSS were slightly higher in the fruit treated with pyrogallol compared with control fruit, whereas the two groups had similar levels of TA.

Total soluble solids and titrable acidity often reflect eating quality [14]. Jiang and Li [15] reported that TTS and TA contents decreased during storage at low temperature. In the present study, TSS and TA tended to decrease over time, with little effect of storage temperature (4 or 25°C).

### Weight loss and respiration rates

**Figure 2:**
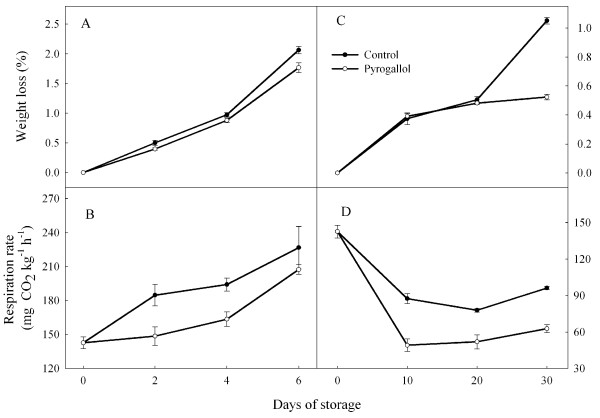
**Effect of pyrogallol on weight loss and respiration rate in ‘Huaizhi’ litchi fruit during storage at 25 (A and C) or 4°C (B and D).** Data are the means ± standard errors of 3 replicates. ● = control, and ○ = pyrogallol.

Weight loss tended to increase over time, and was greater at 25°C than at 4°C (Figure 2). The effect of pyrogallol was generally small, except at the end of a long-term storage, when weight loss was lower in the treated fruit. Respiration increased over time at 25°C, but generally decreased over time at 4°C, and was usually higher at the higher temperature. Pyrogallol typically decreased respiration rate.

Loss of water typically reduced in litchi fruit quality. Jiang and Fu [16] found that the loss of weight of fruit packed in polyethylene bags was mainly due to respiration, and that storage at low temperature slowed this decline. Pyrogallol appeared to reduce weight loss in fruit stored for a long periods at low temperature. Partly this response appeared to be due to a lower respiration rate.

### Phenolic and anthocyanin contents

**Figure 3:**
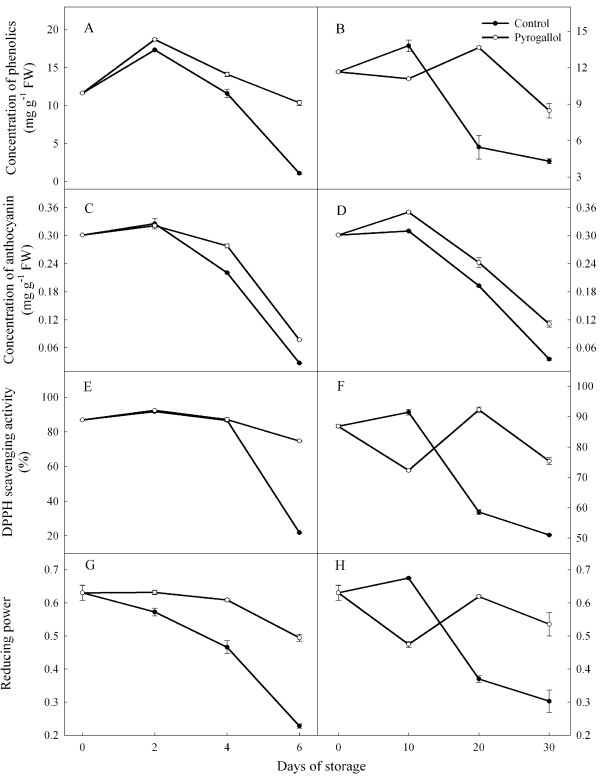
**Effect of pyrogallol on concentrations of phenolics, anthocyanin, DPPH radical scavenging activity and reducing power in ‘Huaizhi’ litchi fruit during storage at 25 (A, C, E and G) or 4°C (B, D, F and H).** Data are the means ± standard errors of 3 replicates. ● = control, and ○ = pyrogallol.

Average phenolic contents rose initially and then declined during storage at ambient temperature (Figure 3). In contrast, values at low temperature were only consistently lower at the end of storage. Average values were higher at 25°C, and higher in the treated fruit. Values of anthocyanin content tended to decrease over time, were similar at the two temperature regimes, but were generally higher in the treated fruit.

The litchi pericarp contains significant amounts of anthocyanins and polyphenols [17]. However, the degradation or the oxidation compounds by PPO or POD results in the pericarp turning brown [4,18]. Anthocyanidin from the hydrolysis of anthocyanins and phenolic compounds acted as possible substrates for POD and PPO [18]. Thus, many physical or chemical methods that inhibit the degradation of anthocyanins and the oxidation of phenolic compounds have been used commercially to slow the browning of litchi fruit during storage [12,13]. In this study, part of the effect of pyrogallol in reducing pericarp browning appeared to be due to the increases in concentrations of phenolics and anthocyanins.

### Peroxidase (POD), polyphenol oxidase (POD) and phenylalanin ammonialyase activities (PAL)

**Figure 4:**
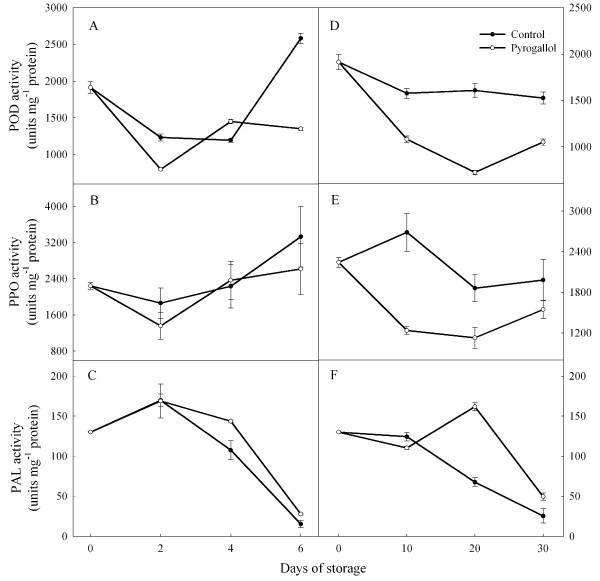
**Effect of pyrogallol on activities of peroxidase (POD), polyphenol oxidase (PPO) and phenylalanin ammonialyase (PAL) in ‘Huaizhi’ litchi fruit during storage at 25 (A, C and E) or 4°C (B, D and F).** Data are the means ± standard errors of 3 replicates. ● = control, and ○ = pyrogallol.

There were no clear trends over time across the different treatment (storage or chemical treatment) on the activities of POD, PPO and PAL (Figure 4). Overall activitie of these enzymes were similar at 4 and 25°C. Activities of these enzymes were generally similar in the control and treated fruit when the fruit were stored at high temperatures. POD and PPO activities tended to be higher and PAL activity lower in the treated fruit stored at low temperatures.

Plants have a network of different antioxidants, such as ascorbate, glutathione, phenolic compounds, anthocyanins and tocopherols. PAL is the key enzyme involved in the biosynthesis of anthocyanins in fruit [19,20]. Zhang et al. [4] reported that the degradation of anthocyanins led to the formation of anthocyanidin, and then POD and PPO catalyzed the degradation of anthocyanidin, resulting in browning of the fruit. In our study, pyrogallol increased the activity of PAL and decreased the activities of POD and PPO. These responses in treated fruit appear to have led to higher average concentrations of anthocyanins and phenolics.

### Membrane permeability and malondialdehyde (MDA)content

**Figure 5:**
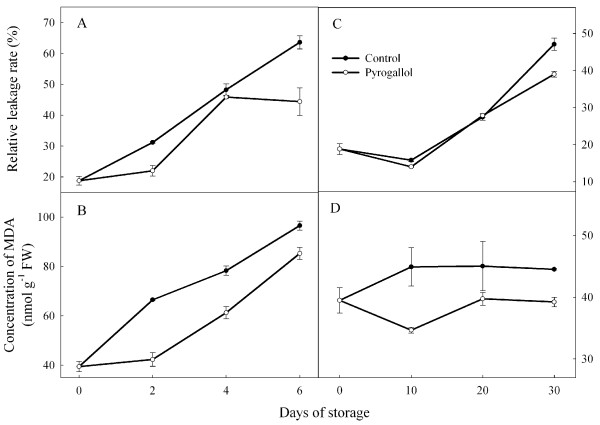
**Effect of pyrogallol on membrane permeability and malondialdehyde (MDA) in ‘Huaizhi’ litchi fruit during storage at 25 (A and C) and 4°C (B and D).** Data are the means ± standard errors of 3 replicates. ● = control, and ○ = pyrogallol.

Average relative leakage tended to increase over time, and was greater at the high temperature than at the low temperature (Figure 5). Pyrogallol treatment reduced leaking compared with the control fruit on days 2 and 4 at 25°C, and on day 30 at 4°C. Average values of MDA increased over time at high temperatures, but were relatively stable at low temperatures (Figure 5). Average values were also higher at the high temperature, and higher in the control fruit than in the treated fruit.

Loss of membrane integrity leading to de-compartmentation of enzymes and substrates [3,21] is involved in browning of the pericarp. Relative leakage rate is an important index of membrane integrity. MDA concentratin reflects the extent of membrane lipid peroxidation caused by oxidation [22,23]. In the present study, pyrogallol decreased MDA accumulation compared with control fruit, whereas the effect on relative leaking rate was smaller. These results suggested a link between browning and the loss of membrane integrity.

### DPPH radical scavenging activity and reducing power

Average values of DPPH scavenging activity and reducing power were similar at the two storage temperatures (Figure 3). Pyrogallol increased DPPH activity compared with control fruit on day 6 at 25°C, and increased reducing power on days 2, 4 and 6. Under low temperature condition, changes in DPPH radical scavenging activity in the control and treated fruit reflected the changes as reducing power. However, there were not consistent differences in these two parameters between the two treatments.

**Figure 6:**
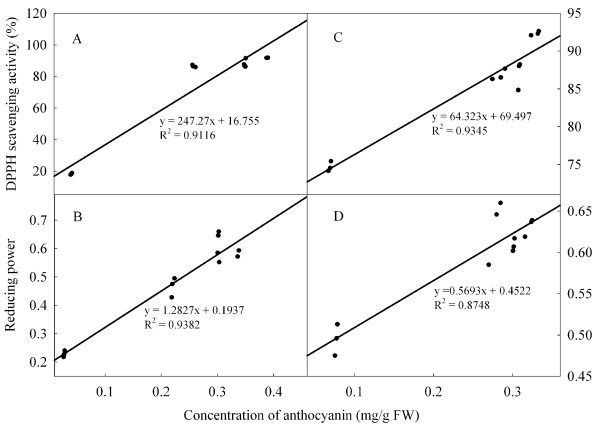
Correlations of concentration of anthocyanins with DPPH radical scavenging activity and reducing power in pericarp of ‘Huaizhi’ litchi fruit at ambient temperature (A and B, control; C and D, pyrogallol).

**Figure 7:**
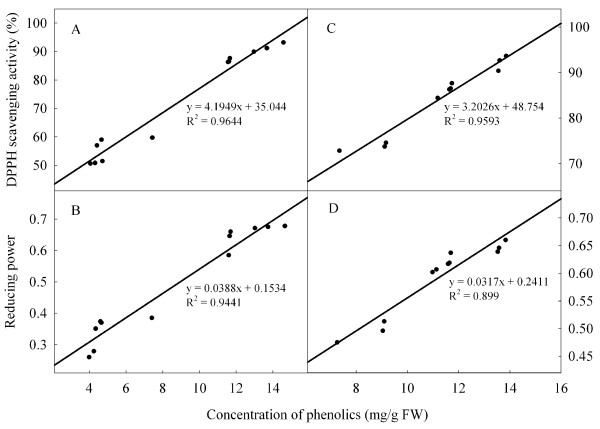
Correlations of concentration of phenolics with DPPH radical scavenging activity and reducing power in pericarp of ‘Huaizhi’ litchi fruit at low temperature (A and B, control; C and D, pyrogallol).

Strong antioxidant activity can help to maintain the red colour of litchi fruit [17]. High contents of anthocyanins and phenolic compounds can also enhance non-enzymatic antioxidant activity (reducing power and free radical scavenging activity) [12]. In this study, there was a significant relationship between the concentrations of anthocyanins or phenolic compounds with reducing power or free radical scavenging activity of litchi fruit during storage (Figures 6 and 7). Overall, pyrogallol reduced the decline in concentrations of anthocyanins and phenolic compounds, and increased reducing power and DPPH scavenging activity.

### Identification and quantification of phenolic compounds

**Figure 8:**
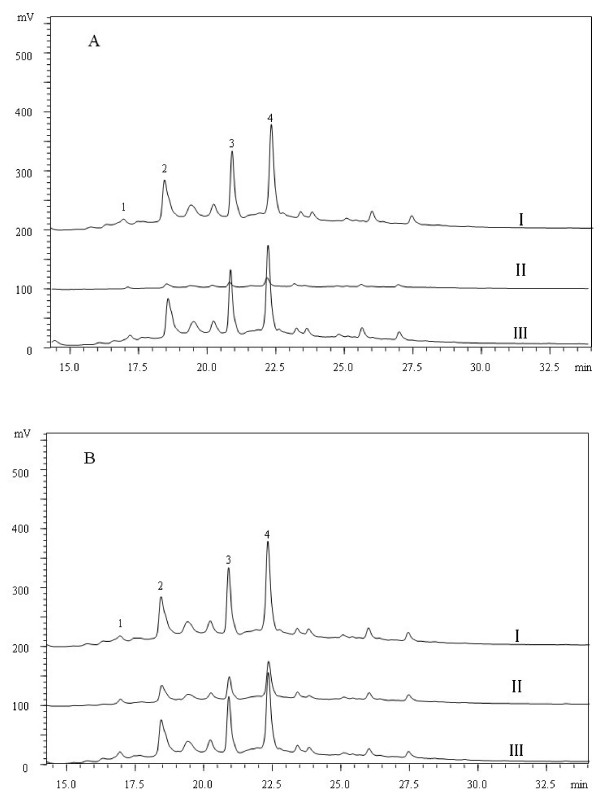
**HPLC profiles of phenolic compounds from the pericarp of ‘Huaizhi’ litchi fruit stored at 4 and 25°C. A**: I, 0 day, II, control fruit and III, treated fruit after 6 days of storage at 25°C and B: I, 0 day, and II, control fruit and III, treated fruit after 30 days of storage at 4°C. 1 = procyanidin **B**1; 2 = catechin; 3 = (−)- epicatechin and 4 = (−)- epicatechin −3-gallate.

Four phenolic compounds, namely procyanidin B1, (+)-catechin, (−)-epicatechin and (−)-epicatechin-3-gallate were identified from the litchi pericarp (Figure 8), with the last three compounds more important. The relative contents of three major compounds were higher in the treated fruit than those in the control fruit.

Phenolics are secondary metabolites that are synthesized during fruit maturation and storage. Monomeric phenolics like (−)-epicatechin and procyanidin A2 present in the pericarp of litchi have a strong antioxidant activity [24]. In this study, (+)-catechin, (−)-epicatechin and (−)-epicatechin-3-gallate were the major phenolics as reported previously [25-28] and were all higher in the treated fruit. These compounds act as free radical-scavengers working as hydrogen or electron doners or assist in metal ion-chelating in litchi pericarp [26,27].

## Materials and methods

### Fruit materials

Fresh mature fruit of litchi cv. Huaizhi were obtained from an orchard in Guangzhou, China. Fruit were dipped for 3 min in 0 (control) or 1 mM pyrogallol, air-dried for 30 min at 25°C, and packed in polyethylene bags (0.03 mm thick and 250 × 200 mm). A concentration of 1 mM of pyrogallol exhibited the best inhibition of browning in a previous experiment. The bags (20 fruit per bag) were fastened with rubber band and stored for 6 days at 25°C, or for 30 days at 4°C. Samples were taken after 0, 2, 4 and 6 days at 25°C, or after 0, 10, 20 and 30 days of storage at 4°C.

### Weight loss, disease incidence and browning

Weight loss was estimated by measuring the changes in fresh fruit weight over time, and expressed as a percentage of initial weight. Disease development was monitored by randomly collecting 30 fruit and recording the percentage showing signs of fungal or bacterial infection. The extent of the total browned area on the pericarp of 30 fruit was accessed using the following scale: 0 = no browning; 1 = slight browning; 2 = <1/4 browning; 3 = 1/4 – 1/2 browning; and 4 = >1/2 browning. The incidence of browning was calculated as ∑ (browning scale × number of fruit in each class) / (number of total fruit × highest browning scale) × 100.

### Total soluble solids and titratable acidity

Pulp (20 g) from 3 replicates of 6 fruit from each treatment was homogenized in a grinder and centrifuged for 20 min at 15 000 × *g*. The supernatant was analysed for total soluble solids using a hand refractometer (J1-3A, Guangdong Scientific Instruments) and titratable acid using titration with 0.1 M NaOH.

### Respiration

Respiration rate was measured by infrared gas analysis. Three replicates of 9 fruit from each treatment were weighed before being sealed in a 2.4-L container at 25°C. Increased CO_2_ concentration in the container was monitored using an infrared gas analyzer (Li-6262 CO_2_/H_2_O analyzer, LI-COR, Inc. USA). Respiration was expressed as mg CO_2_ per hour per g FW.

### Membrane permeability

Membrane permeability, expressed by relative leakage rate, was determined according to the method of Duan et al. [6]. Pericarp discs were removed with a cork borer (10 mm in diameter) from the equatorial region of 30 fruit. The discs (about 2 g) were rinsed twice, incubated in 25 mL of 0.3 M mannitol at 25°C, and then shaken for 30 min. Electrolyte leakage was determined with a conductivity meter (Model DDS-11A, Shanghai, China). Another batch of discs were boiled for 15 min in 25 mL of distilled water and cooled to 25°C to assess total electrolytes. Relative leakage was expressed as a percentage of the total electrolytes, and then calculated using the following formula: (electrolyte / total electrolytes) × 100.

### Total anthocyanin and phenolic contents

Pericarp tissues (5 g) from 30 fruit were blanched with 200 mL of 0.1 M HCl. The extract solution (5 mL) was diluted in 25 mL of 0.4 M KCl-HCl buffer (pH 1.0), and 25 mL of 0.4 M citric acid-Na_2_HPO_4_ buffer (pH 4.5). A spectrophotometer (UVmini-1240, Shimadzu Corp, Japan) was used to measure total anthocyanin content at 510 nm. Total anthocyanin content was expressed as cyanidin-3-glucoside equivalent on a FW basis.

For total phenolic content, pericarp tissues (5 g) from 30 fruit were extracted for 2 h in 100 mL methanol containing 0.1 M HCl at 25°C. The extraction solution was filtered and the filtrate was collected for phenolic content determination. Phenolic content was measured by the Prussian blue assay of Price and Buttler [29] and expressed as gallic acid equivalent on a FW basis.

### 2,2-Diphenyl-1-picrlhydrazyl radical scavenging activity

Pericarp tissue (5 g) from 30 fruit was ground with liquid nitrogen and extracted for 30 min in 30 mL of methanol containing sodium metabisulphite (0.5%). The homogenate was filtered through four layers of cheesecloth while the residue was added to 20 mL of the same extraction solution for two repeated extractions. The collected filtrate was centrifuged for 20 min at 20,000 × *g* at 4°C. Scavenging 2,2-diphenyl-1-picrlhydrazyl (DPPH) radical activity was assessed by the method of Larrauri [30] with some modifications. Briefly, litchi methanol extract solution (0.1 mL) at various concentrations was mixed with 2.9 mL of 0.1 mM DPPH-methanol solution. After the mixed solution was incubated for 30 min at 25°C in the dark, absorbance at 517 nm was measured. The control was carried out with water instead of the tested sample, while methanol instead of DPPH was used for the blank. The DPPH radical scavenging activity (%) of the tested sample was calculated as [1 − (absorbance of sample − absorbance of blank) / absorbance of control)] × 100.

### Reducing power

Reducing power was determined according to the method of Oyaizu [31]. An aliquot (0.25 mL) of methanol extract solution at various concentrations was mixed with 2.5 mL of 0.2 M sodium phosphate buffer (pH 6.6) and 2.5 mL of 1% potassium ferricyanide. The mixture was then incubated for 20 min at 50°C. After 2.5 mL of 10% trichloroacetic acid (w/v) were added, the mixture was centrifuged at 650 × *g* for 10 min. An aliquot (5 mL) of the upper layer was collected and mixed with 5 mL of distilled water and 1 mL of 0.1% ferric chloride. The absorbance at 700 nm was measured, with higher absorbance indicating a higher reducing power.

### Lipid peroxidation

Lipid peroxidation was evaluated by measuring malondialdehyde (MDA) content by the method of Duan et al. [12]. Pericarp tissues (5 g) from 30 fruit were homogenized in 25 mL of 50 g/L (w/v) trichloroacetic acid (TCA) and centrifuged for 10 min at 4000 × *g*. An aliquot (1 mL) of the supernatant was mixed with 3 mL of 0.5% 2-thiobarbituric acid (TBA) in 10% TCA, incubated for 20 min in boiling water, cooled quickly, and centrifuged for 10 min at 4000 × *g*. The absorbance of the supernatant was measured at 532 nm and corrected for non-specific turbidity by subtracting the absorbance at 600 nm. The concentration of MDA was calculated with an extinction coefficient of 155 mM^-1^ cm^-1^ and expressed as nmol/g FW = 258 (*A*_*532*_*- A*_*600*_).

### Peroxidase, polyphenol oxidase and phenylalanin ammonialyase activities

Pericarp tissues (4.0 g) from 30 fruit were homogenised with 12 mL of 50 mM potassium phosphate buffer (pH 7.0) containing 1% (w/v) polyvinylpyrrolidone (insoluble). After centrifugation for 15 min at 10,000 × *g* at 4°C, the supernatant was collected and used as the crude enzyme extract. POD activity using guaiacol as substrate was assayed by the method of Zhang et al. [4] in a reaction mixture (3 mL) containing 0.05 mL enzyme solution, 2.75 mL of 50 mM phosphate buffer (pH 7.0), 0.1 mL of 1% H_2_O_2_ and 0.1 mL of 4% guaiacol. The increase in absorbance at 470 nm due to guaiacol oxidation was recorded for 2 min. One unit of enzymatic activity was defined as the amount of the enzyme that caused a change of 0.01 in absorbance per minute. PPO activity was assayed according to the method of Jiang and Fu [16] by measuring the oxidation of 4-methylcatechol. The increase in absorbance at 410 nm was recorded for 3 min. One unit of enzymatic activity was defined as the amount that caused a change of 0.001 in absorbance per minute.

For phenylalanin ammonialyase (PAL) activity, pericarp tissue (5 g) from 30 fruit were homogenized in 20 mL of 0.1 M sodium borate buffer (pH 8.0) containing 0.5 g of polyvinylpyrrolidone (insoluble), 5 mM β-mercaptoethanol and 2 mM EDTA. The homogenate was centrifuged for 20 min at 19,000 × *g* at 4°C and the supernatant collected for enzyme assay. PAL activity was determined by incubating 0.1 mL of enzyme extract solution and 2.9 mL of 0.1 M sodium borate buffer (pH 8.0) containing 3 mM L-phenylanine for 1 h at 37°C. The increase in the absorbance at 290 nm was measured spectrophotometrically, due to the formation of transcinnamate. One unit of enzyme activity was defined as the amount that caused an increase of 0.01 in the absorbance per hour.

Protein content was determined by the method of Bradford [32], with bovine serum albumin used as a standard.

### Phenolic compounds

Pericarp tissue (2 g) from 30 fruit were resolved in 40 mL of 60% ethanol at 30°C for 30 min using an ultrasonic cleaner (40 kHZ, SB-5200DTD, Xinzhi Biotech Co., Ningbo, China). After extraction, the solution was filtered through 13 mm and 0.45 μm PVD membranes (Shanghai ANPEL Scientific Instruments Co. Ltd., Shanghai, China). Separation, analysis and quantification of phenols were done in a high performance liquid chromatograph (HPLC) (Shimadzu LC-20 AT, Shimadzu Corporation, Japan), coupled with a Vydac C18 column (218 TP, 250 × 4.6 mm, 5 μm of particle size, Sigma-Aldrich, St. Louis, MO, USA) and a SPD-10A UV–VIS detector using the method of Lin et al. [33]. The samples were eluted with a gradient system consisting of solvent A (0.1% formic acid) and solvent B (methanol) used as the mobile phase at a flow rate of 1 mL/min. The gradient elution program was as follows: 0–5 min, 10% B; 5–35 min, 10–100% B; 35–40 min, 100% B; and 40–45 min, 10% B. The temperature of the column was 25°C, the injection volume was 15 μl, and the chromatogram was recorded at 280 nm. Identification of individual phenols was estimated on the basis of their retention times.

### Data analyses

The experiments were arranged in completely randomized design. Data were presented as the means and standard errors (SE). The linear regressions were made between the concentrations of anthocyanins or phenolic compounds with reducing power or free radical scavenging activity.

## Conclusions

This study showed that pyrogallol reduced generally the incidence of disease and browning in litchi fruit during storage, and this chemical treatment also reduced respiration rate and leakage from the cellular membranes, and maintained high levels of DPPH radical scavenging activity and reducing power. Furthermore, pyrogallol suppressed the activities of POD and PPO, enhanced the activity of PAL, and maintained high concentrations of anthocyanins and phenolics. Overall, pyrogallol helped to maintain overall quality attributes and reduced quality-related physiological and biochemical activities. It was suggested that the application of pyrogallol could be beneficial in the browning and decay control of litchi fruit. To some extent, we consider that it might be suitable for treatment of fruit for short storage, transport or distribution (e.g. 2 or 4 days at ambient temperature).

## Competing interests

The authors declare that they have no competing interests.

## Authors’ contributions

Guoxing Jing was the major contributor to acquisition of data, analysis and manuscript preparation. Hua Huang partly participated in experiments and data analysis. Bao Yang made a substantial contribution to experimental design and data analysis. Jianrong Li and Xiaolin Zheng participated in the design of the study and manuscript revision. Yueming Jiang made a significant contribution to experimental design, data analysis, and manuscript revision. All authors read and approved the final version of the manuscript.
